# Minimizing Postoperative Scars in Epicanthoplasty: A Concise Review

**DOI:** 10.1111/jocd.70603

**Published:** 2025-12-10

**Authors:** Fredrik A. Fineide, Richard C. Allen, Elin Bohman, Kim A. Tønseth, Tor P. Utheim, Ayyad Z. Khan

**Affiliations:** ^1^ Department of Plastic and Reconstructive Surgery Oslo University Hospital Oslo Norway; ^2^ Department of Medical Biochemistry Oslo University Hospital Oslo Norway; ^3^ The Norwegian Dry Eye Clinic Oslo Norway; ^4^ Department of Computer Science Oslo Metropolitan University Oslo Norway; ^5^ Department of Ophthalmology Østfold Hospital Trust Moss Norway; ^6^ Department of Ophthalmology, Dell Medical School University of Texas at Austin Austin Texas USA; ^7^ Oculoplastic and Orbital Services St. Erik Eye Hospital Stockholm Sweden; ^8^ Division of Eye and Vision, Department of Clinical Neuroscience Karolinska Institutet Stockholm Sweden; ^9^ Division of Surgery and Specialized Medicine Oslo University Hospital Oslo Norway; ^10^ Institute for Clinical Medicine, Faculty of Medicine University of Oslo Oslo Norway; ^11^ Department of Ophthalmology Sørlandet Hospital Trust Arendal Norway; ^12^ Department of Ophthalmology Oslo University Hospital Oslo Norway; ^13^ Department of Ophthalmology Stavanger University Hospital Oslo Norway; ^14^ Department of Ophthalmology Vestre Viken Hospital Trust Drammen Norway; ^15^ Department of Ophthalmology Vestfold Hospital Tønsberg Tønsberg Norway

## Abstract

**Background:**

The epicanthal fold is a fibromuscular skin fold covering the medial aspect of the eye. Upper double eyelid blepharoplasty and epicanthoplasty have become the most frequently performed cosmetic surgeries in Asia. However, many surgeons have expressed concern for hypertrophic scarring following epicanthoplasty. Therefore, exploring scar‐minimizing techniques should interest surgeons performing epicanthoplasty, especially since most candidates for this type of surgery are more susceptible to hypertrophic scarring due to genetic factors.

**Objectives:**

This review aims to contribute to reducing post‐epicanthoplasty scarring by presenting a synopsis of the existing medical literature on scar‐minimizing strategies for medial epicanthoplasty.

**Methods:**

PubMed and EMBASE searches were conducted on October 25, 2025, and following screening, 85 publications were included.

**Results:**

As novel techniques have increasingly focused on tension release within the epicanthal fold, most procedures are now well‐tolerated with a low recurrence rate and a high degree of patient satisfaction.

**Conclusions:**

While numerous techniques have been described, no single method suits all cases. Based on trends in the literature, the skin redraping technique appears to be a versatile option with limited scarring in diverse scenarios, though this inference is not supported by randomized or prospective comparative data. Prospective, randomized studies are needed to compare the efficacy and cosmetic outcomes of different approaches for different degrees of epicanthal folds.

## 1 Introduction

**FIGURE 1:**
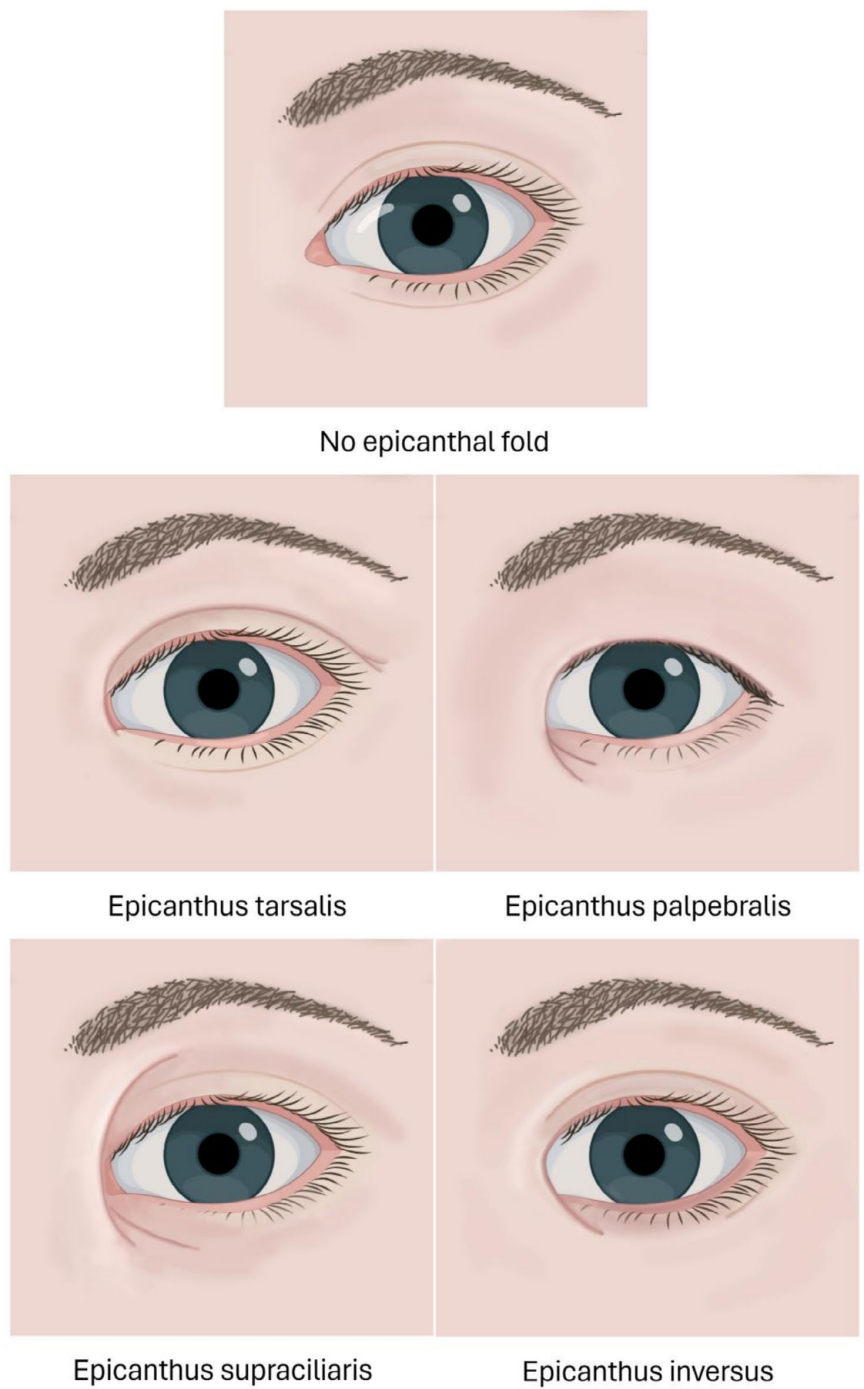
The four types of epicanthal folds described by Duke–Elder [2]. Illustration by Tanya Cross.

The epicanthal fold is a semilunar, medial extension of the eyelid skin wholly or partially obscuring the lacrimal lake. Duke–Elder described four types of epicanthal folds (Figure 1) [1]:

1. *Epicanthus supraciliaris*: The fold originates in the region of the eyebrow and runs toward the lacrimal sac.
2. *Epicanthus palpebralis*: The folds originates above the tarsus of the upper eyelid and extends toward the inferior part of the orbit.
3. *Epicanthus tarsalis*: The folds originates from the tarsal fold and fades into the medial canthus.
4. *Epicanthus inversus*: The folds originates from the lower eyelid, runs superiorly in a crescentic fashion, fading into the upper eyelid. *Epicanthus inversus* is associated with blepharophimosis, ptosis and epicanthus inversus syndrome, an autosomal dominant condition secondary to mutations in the FOXL2 gene [3].

The presence of an epicanthal fold is common during embryological development. While epicanthal folds recede within the first years of life in most non‐Asians, they are common in Asian ethnicities [4]. Epicanthal folds cause a wider distance between the canthi and a shorter palpebral fissure. Although traditionally considered an attractive part of Asian periocular features, the changing societal beauty standards have made epicanthoplasty the most commonly performed procedure in Asian cosmetic surgery [5]. One important exception to it being a cosmetic procedure is medically indicated upper eyelid blepharoplasty in Asians, in which epicanthoplasty should be performed concurrently to avoid an epicanthal fold exaggeration caused by the removal of upper eyelid tissue.

Previously, it was thought that epicanthal folds only consisted of redundant skin. Histological studies, however, have uncovered that the folds contain bands of fibromuscular tissue connecting the upper and lower pretarsal orbicularis muscles [6, 7]. To release this connection, undermining between the skin and underlying orbicularis has been considered important in epicanthoplasty [5]. Considering that most candidates for this type of surgery are more susceptible to hypertrophic scarring due to genetic factors [8], exploring scar‐minimizing techniques should interest every surgeon performing epicanthoplasty. This review aims to contribute to reducing post‐epicanthoplasty scarring by presenting a synopsis of the medical literature published on scar‐minimizing strategies for medial epicanthoplasty.

## 2 Methods

**FIGURE 2:**
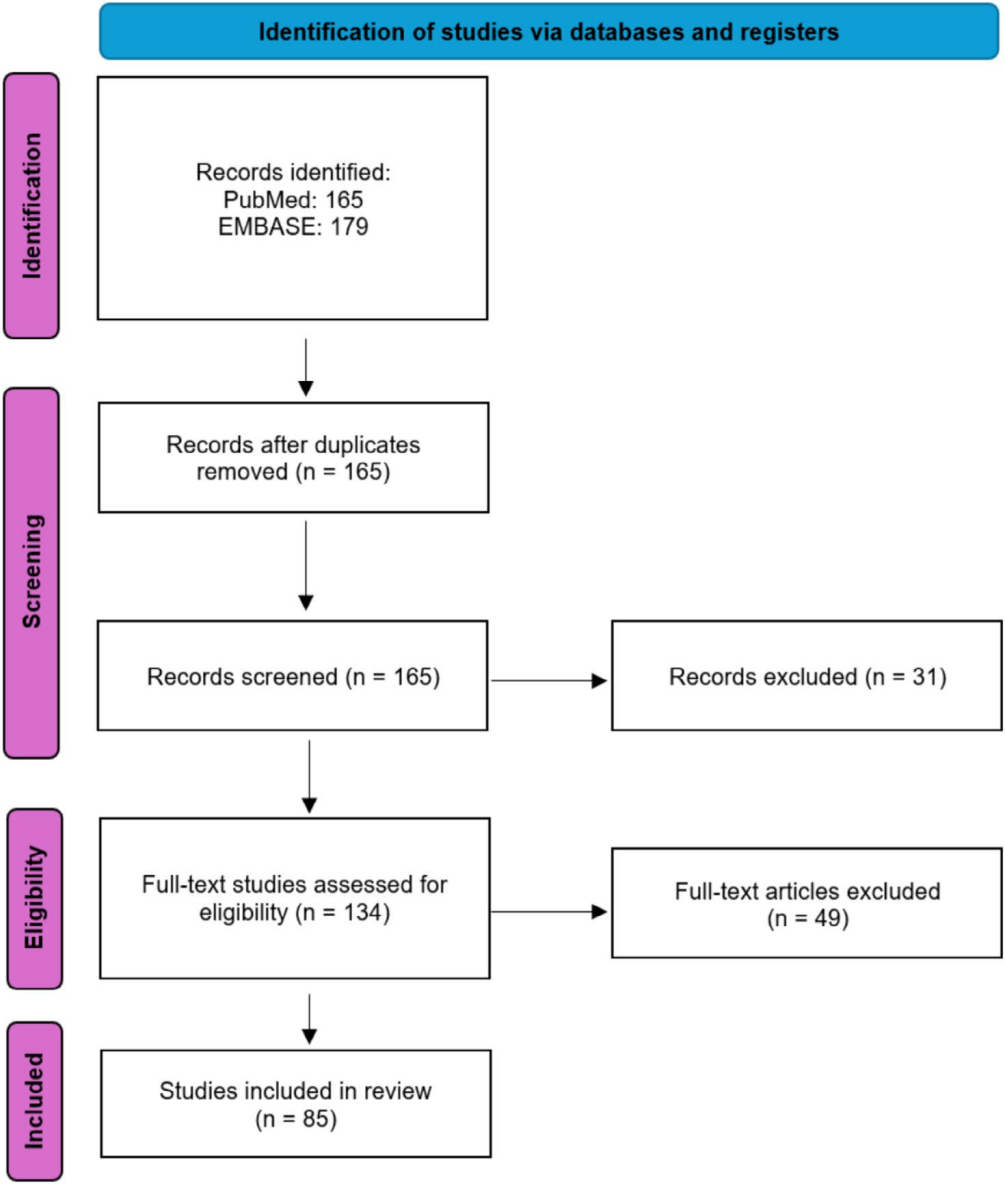
A PRISMA flow diagram of study identification, inclusion, and exclusion.

The PubMed and Ovid EMBASE databases were searched on October 25, 2025, using the search word “epicanthoplasty,” no additional search terms or keywords were used. The search identified 165 and 179 records in PubMed and EMBASE, respectively. The records were screened manually based on title and abstract. Articles were excluded according to predetermined criteria, i.e., publications not discussing scarring after medial epicanthoplasty and publications primarily addressing reconstruction efforts for established post‐epicanthoplasty scars. Also excluded were patents, dissertations, and articles published in languages other than English. Following this screening, 134 records remained eligible for full‐text evaluation. After the full‐length assessment, 85 English‐language publications investigating post‐operative scarring in medial epicanthoplasty were included in the final qualitative synthesis (Figure 2).

## 3 Results

A total of 85 publications investigating postoperative scarring following medial epicanthoplasty were included in the qualitative synthesis (Table S1). These studies were published between 1984 and 2025. Twelve studies focused on the correction of congenital anomalies, whereas the remainder evaluated cosmetic epicanthoplasty procedures. The included studies encompassed a wide range of surgical techniques. Among them, four described the skin redraping technique, five discussed variations of a subcutaneous approach, four evaluated *Y*‐*V* epicanthoplasty, and 23 publications reported on *Z*‐epicanthoplasty. Among the *Z*‐epicanthoplasty publications, 17 described excision of the orbicularis oculi muscle [9, 10, 11, 12, 13, 14, 15, 16, 17, 18, 19, 20, 21, 22, 23, 24, 25], seven reported release of the orbicularis oculi from the surrounding tissues [26, 27, 28, 29, 30, 31, 32], five mentioned anchoring to the deeper tissues or periosteum [10, 11, 14, 32, 33], and three performed plication of the medial canthal ligament [12, 13, 24]. The remaining 26 publications described alternative or combined methods (the “other” category). Across these studies, a substantial heterogeneity of technique was observed, reflecting the absence of standardized protocols. Comparative studies were limited but provided useful insight: Ten compared the cicatricial outcome following different surgical approaches, eight of these compared different incisional methods [34, 35, 36, 37, 38, 39, 40, 41], and two evaluated surgical outcomes depending on the excision of deeper tissues [42, 43]. The surgical approach was determined based on surgeon preference [34, 41], the severity of the epicanthal folds [35, 36], or was not reported [37, 38, 39, 40]. Overall, recent literature demonstrates a progressive shift toward tension‐release techniques, with newer procedures showing lower recurrence rates and improved scar profiles. However, due to the lack of prospective comparative trials, the relative efficacy of these methods remains uncertain.

## 4 Discussion

### 4.1 Origins

Understanding of an entity's evolutionary, embryological, physiological and anatomical properties can be valuable when attempting a surgical correction. The origin, cause, and composition of the epicanthal folds vary across the different types and are still somewhat unclear. Early epicanthoplasty techniques incorrectly assumed that tightening the dermis alone could correct the fold. This caused traction across incisions, leading to hypertrophic scars and secondary folds. Novel approaches address deeper tissues, including the orbicularis oculi muscle, the medial canthal ligament, and the fibrous tissue between the two. It has been hypothesized that the medial epicanthal fold results from: orbicularis oculi muscle fiber malposition [44]; orbicularis oculi muscle skin tension [45]; a combination of superficial fibers of the medial canthal ligament in conjunction with the orbicularis oculi muscle and excessive fibrofatty tissue [31]. An evolutionary theory of the medial epicanthal folds has been proposed, describing environmental factors leading to excessive frowning that causes hypertrophy of the orbicularis oculi and depressor supercilii muscles. This causes sheer, tensional, and compressional stresses, leading to the contraction and subsequent atrophy and fibrosis of the orbicularis oculi muscle [46].

### 4.2 Preoperative Considerations

Incision placement is a vital part of pre‐operative planning. The included studies exhibited significant heterogeneity in placement and incisional degree. In Asian populations, medial epicanthoplasty is often performed simultaneously as double eyelid surgery to establish a supratarsal crease. Many surgeons, however, are reluctant to perform concurrent medial epicanthoplasty due to the risk of excessive scarring [4]. This omission may lead to an unfortunate result: The accentuation of the epicanthal fold due to increased lateral tension [7]. When both procedures are performed simultaneously, the incision line of the medial epicanthoplasty should be either a continuation of the newly established supratarsal crease or hidden within the subciliary line [47]. The thin eyelid skin is somewhat forgiving regarding scar formation, while the thicker, more medial nasal skin scars easily [4]. Therefore, incisions medial to the medial canthus as in some earlier approaches might predispose to greater scarring.

### 4.3 Surgical Techniques

#### 4.3.1 Skin Redraping Technique

**FIGURE 3:**
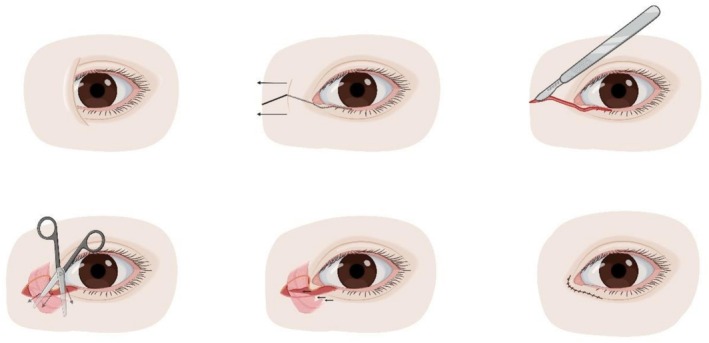
The skin redraping technique. Top row: Preoperative appearance; incision design; incision. Bottom row: Skin flap dissection; orbicularis muscle release and resection; postoperative appearance.

Oh et al. published the skin redraping technique in 2007. The original approach consisted of a curvilinear incision extending from the location of the new medial epicanthus laterally along the subciliary margin of the lower eyelid (Figure 3) [48]. A later procedure aimed to address a tendency for hooding consists of an incision running inferomedially as an extension of the double eyelid line in addition to a lower *V*‐shaped incision with excision of excess dermis [49].

The skin redraping technique is versatile and has been used with and without ancillary procedures [50, 51, 52]. Moreover, it has proven beneficial for blepharophimosis, ptosis and epicanthus inversus syndrome [53], and congenital telecanthus [54]. The ability to adjust the degree of correction through an individualized degree of orbicularis oculi excision, medial canthal ligament plication and periosteal anchoring combined with no incisions over the nasal skin makes this technique extremely versatile. Importantly, these features allow for correcting the epicanthal fold and skin closure without significant postoperative tension on the wound, thus resulting in healing without significant scarring.

#### 4.3.2 Subcutaneous Epicanthoplasty

**FIGURE 4:**
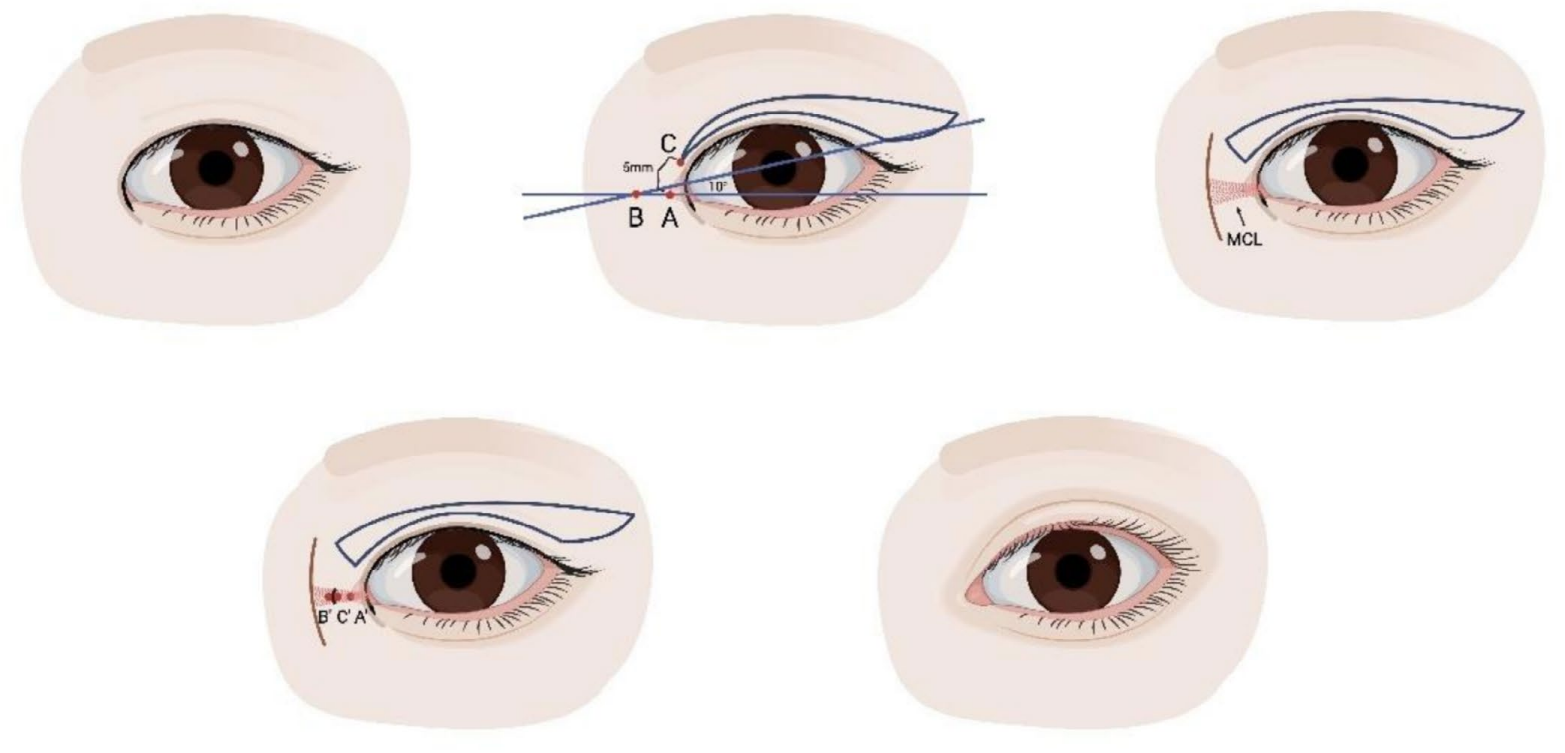
Subcutaneous epicanthoplasty. Top row: Preoperative appearance; incision design; dissection through medial eyelid crease incision. Bottom row: Anchoring suture; postoperative appearance.

Subcutaneous epicanthoplasty was initially described for the correction of epicanthus tarsalis, palpebralis and supraciliaris in pediatric patients [1]. The authors described a medial extension of the upper blepharoplasty incision over the crest of the epicanthal fold, excision of the orbicularis oculi muscle and anchoring of the dermis to the periosteum (Figure 4). This technique effectively eliminates extensive epicanthal folds; however, since the incision is extended over the epicanthal crest, it may result in scarring and is more extensive than necessary to correct the epicanthal fold. Moreover, since the blepharoplasty and epicanthoplasty incisions are continuous, upper eyelid movement may transmit tension, further exacerbating scar formation [55]. To address this, the authors presented a modified, subcutaneous approach in conjunction with upper blepharoplasty or ptosis repair [56].

In the late 1980s, Lee et al. developed anchor epicanthoplasty, correcting epicanthal folds in combination with double eyelid blepharoplasty [45]. The preseptal portion of the orbicularis oculi muscle, superficial fibers of the medial canthal ligament, and underlying adipose and connective tissue were excised and the medial portion of the incision anchored to deeper tissues.

Disruption of the angular or infratrochlear vessels has been described as common with these approaches, resulting in sometimes difficult to control bleeding. Since there is no incision medial to the medial canthus, scarring of the medial epicanthal region is avoided. This approach is suitable for mild to moderate epicanthal folds in Asians but not for more pronounced cases, epicanthus inversus or blepharophimosis. Its disadvantages include technical difficulty and risk of active bleeding.

#### 4.3.3 *Y‐V* Epicanthoplasty

**FIGURE 5:**
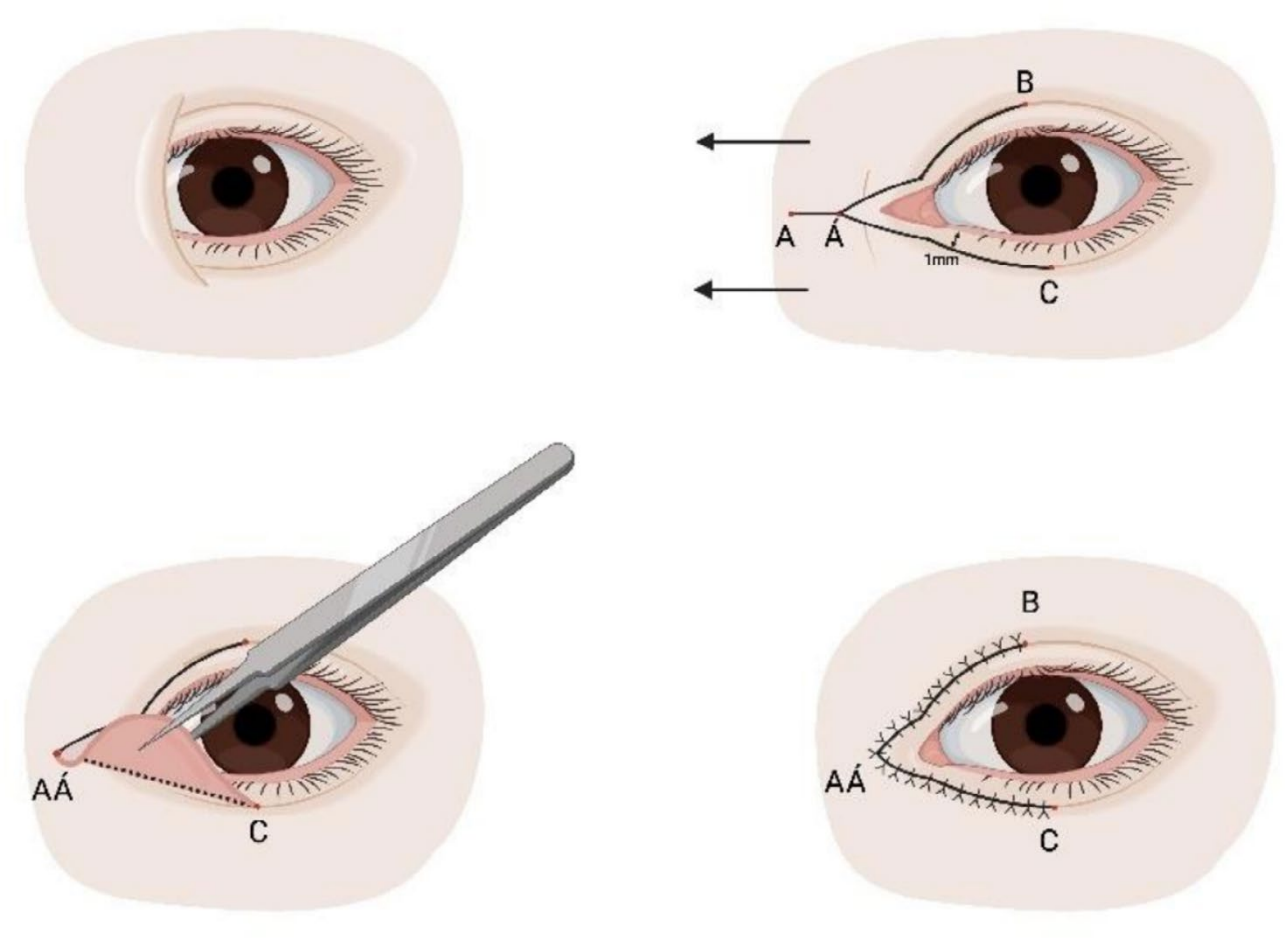
*Y‐V* epicanthoplasty. Top row: Preoperative appearance; incision design. Bottom row: Flap resection; postoperative appearance.

Developed in the early 20th century [57], early approaches involved a horizontally aligned, *Y*‐shaped incision originating in the midline over the nasal dorsum, bifurcating and extending over the superior and inferior eyelids (Figure 5) [58]. The procedure involved extensive dissection and release of the medial canthal ligament and the superior and inferior tarso‐orbital fasciae to facilitate nasal displacement of the medial canthus and consequent periosteal anchoring. It resulted in noticeable scarring. Later modifications were far less extensive, with no incision over the nasal dorsum, but instead centered around the medial canthus extending supraciliary and subciliary, including partial excision of orbicularis oculi and periosteal anchoring [59, 60, 61], or plication of the medial canthal ligament [62]. The main disadvantage remains scar formation.

#### 4.3.4 *Z*‐Epicanthoplasty

**FIGURE 6:**
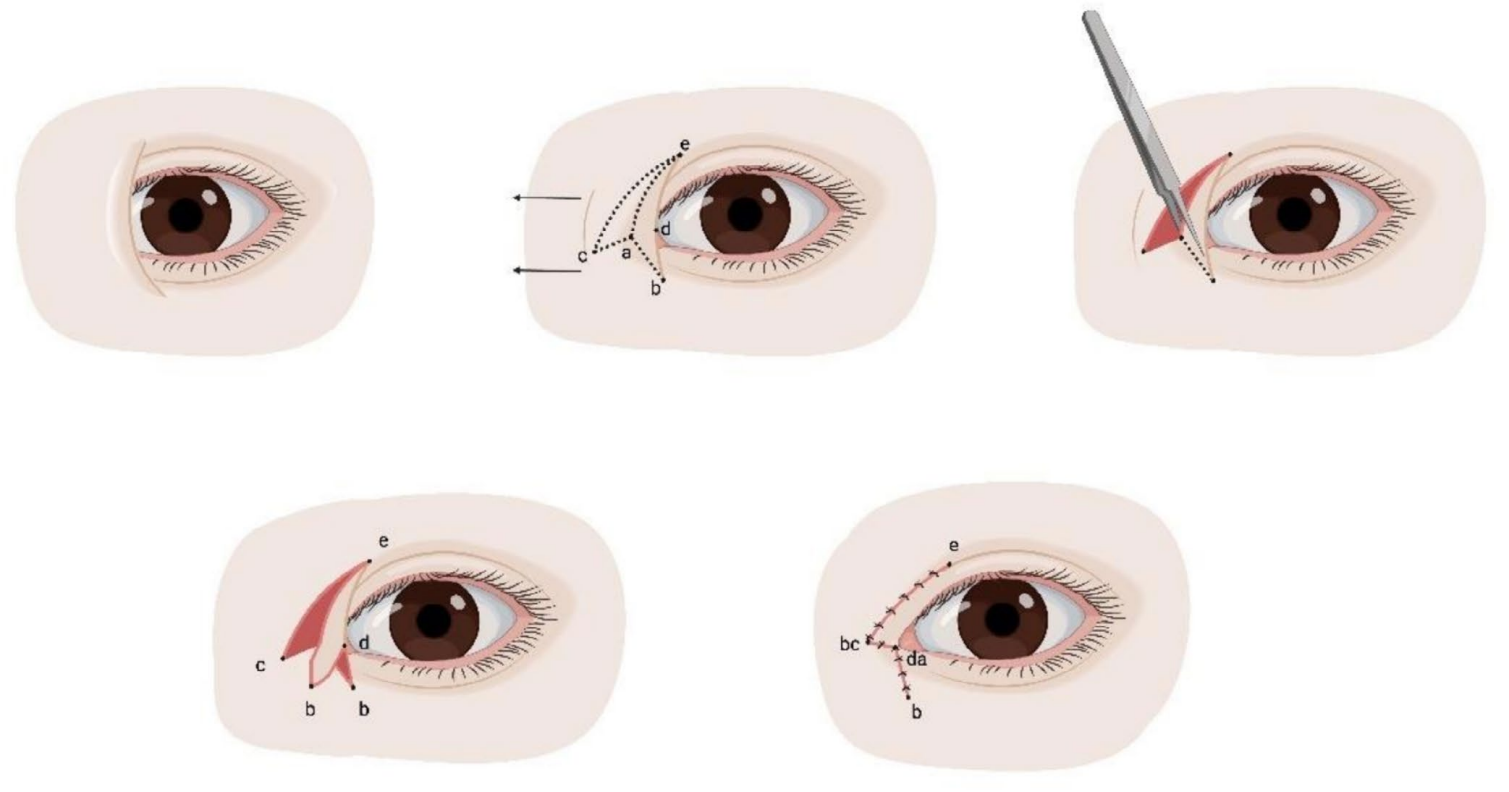
*Z*‐epicanthoplasty. Top row: Preoperative appearance; incision design; triangular resection. Bottom row: Flap is lifted, rotated and sutured; postoperative appearance.

*Z*‐plasty is a transposition flap technique often employed in plastic and reconstructive surgery to release contractures and revise scars [63, 64, 65]. Lessa and Sebastiá presented an approach with nasal transfixation of the medial canthal ligaments using a steel wire with *Z*‐plasty performed on the resulting “dog ears” [9]. While effective, there was potential for refinement, which was realized by Park (Figure 6) [10]. The procedure was performed alongside double eyelid blepharoplasty. It included subcutaneous dissection, transposition of a myocutaneous flap containing the orbicularis oculi muscle, and fixation to deeper soft tissues. A tendency to develop a pretarsal double fold due to a redundancy of skin was noted and a modified approach with a slight change in incision angle and medial canthal tendon plication was proposed [11]. Further modifications included single *Z* incision [13, 15, 16, 19, 30], double *Z* incision [27], and root *Z*‐epicanthoplasties [31]. The *Z*‐epicanthoplasty procedure is the most published and likely the most commonly performed surgical intervention regarding aesthetic correction of the Asian epicanthal fold [66, 67]. Many variations and adaptations exist, and this procedure effectively corrects mild to more severe cases with low recurrence. While the rate of hypertrophic scarring has become relatively low, scar formation and anchoring suture granulomas remain the principal complaints.

#### 4.3.5 Comparative Studies

Among the included comparative studies, most authors recommend *Y‐V* plasty or *Z*‐plasty for moderate epicanthus and *V‐W* plasty or the Mustardé technique for more severe cases [34, 35, 36]. The release or excision of the orbicularis oculi muscle results in less tension in the medial canthal region, leading to less scarring and greater patient satisfaction [42, 43]. There is insufficient evidence to conclude what surgical approach should be used for which type of patient due to the scarcity of studies and the methodology of the few studies performed. One study found that *Z*‐plasty results in less scarring than *Y‐V* plasty and the Mustardé technique [36], while another found contrary evidence with less scarring following *Y‐V* plasty than *Z*‐plasty [39]. *Z*‐plasty might cause less scarring than redraping when correcting epicanthus palpebralis, while redraping might result in greater smoothness when used for epicanthus tarsalis [38]. In summary, the more invasive or comprehensive approaches like *Y‐V* plasty, *V‐W* plasty and the Mustardé technique allow for a greater degree of correction but might also predispose patients to a higher amount of scarring (Table 1). Likewise, the use of anchoring sutures or wires may reduce the recurrence rate but might also entail a more comprehensive procedure and thus increased risk of scar and granuloma formation. Prospective, randomized studies are required to further examine these important questions.

**TABLE 1:** Summary of the most common surgical techniques.

Technique	Indication by epicanthal fold severity	Degree of scarring
Skin redraping	Mild to severe	Mild
Subcutaneous epicanthoplasty	Mild to moderate	Moderate
*Y‐V* epicanthoplasty	Moderate to severe	Moderate to severe
*Z*‐epicanthoplasty	Mild to moderate	Mild to moderate
*V‐W* plasty	Moderate to severe	Moderate
Mustardé	Severe	Moderate

### 4.4 Intraoperative Considerations

Several intraoperative decisions and approaches may influence the degree of postoperative scarring, such as interrupted or running sutures and the choice of suture materials. There are, however, no studies evaluating these factors in medial epicanthoplasty. We recently performed a similar review of the literature on upper blepharoplasty [68]; however, only one included study asked this pertinent research question [69].

Other considerations that may impact postoperative scarring following medial epicanthoplasty include medial canthal ligament plication, periosteal anchoring, flap design or flapless approach, the degree of dissection, and the release or excision of the orbicularis oculi muscle.

Periosteal anchoring and plication of the medial canthal ligament can be performed in more severe cases and is compatible with most approaches. If performed, the anchoring sutures should be placed as deep as possible to minimize the risk of skin erythema and suture granulomas. Nonabsorbable sutures should be used for anchoring and plication to minimize the risk of recurrence of the epicanthal fold.

We believe it is imperative to minimize the degree of surgical incisions medial to the medial canthus and that incisions including the thicker, more scar‐prone nasal skin should be avoided.

Recent evidence shows that a fibromuscular band crossing the medial canthus, partially attached to the dermis, contributes to tension formation [6, 7]. Indeed, several articles described the spontaneous regression of the fold upon the release between muscle and skin. We believe that this release is the most crucial aspect of epicanthoplasty as it ensures tension‐free closure of the dermis, minimizing the risk of recurrence, webbing, and scarring. Additional surgical steps such as plication, anchoring and excision of the orbicularis oculi muscle should depend on the degree of the epicanthal fold, the degree of correction the patient desires and the aesthetic sense of the surgeon.

### 4.5 Methodological Considerations, Limitations, and Future Perspectives

Most novel approaches describe a low degree of scarring and high patient satisfaction. Comparing the degree of scarring across studies is complicated by the heterogeneous reporting of methods and the lack of standardization.

We could not identify any studies evaluating the impact of suturing techniques or the choice of suture size or materials. We believe these factors might impact the wound healing process and the final cicatricial results. Prospective, randomized studies are needed to evaluate this important research question.

Several of the included studies have reported using intralesional steroid injections combatting hypertrophic scarring (Table S1), and two studies included steroid injections as part of the routine postoperative treatment [70, 71]. Only three studies reported the application of steroid ointment as part of the postoperative routine [56, 72, 73], and no studies evaluated the effect of steroid ointment on scarring against an untreated control group. There are a few reports of using intralesional 5‐fluorouracil for periocular hypertrophic scarring [74], although none after cosmetic epicanthoplasty. Further, prospective, randomized studies are warranted examining the effects of these postoperative scar modification techniques to minimize the need for revisional procedures and increase patient satisfaction.

## 5 Conclusion

A greater understanding of the anatomical and histological basis of the epicanthal fold has led to the development of novel techniques. Consequently, scar formation has greatly decreased over the past few decades. While numerous surgical techniques for eliminating or reducing the appearance of the epicanthal fold have been described, no one technique is suitable for all cases. Although not supported by comparative trials, the aggregate of reports suggests that skin redraping is versatile, owing to tension release without nasal skin incisions and the ability to tailor orbicularis management and periosteal anchoring across a range of severities. Nevertheless, prospective, randomized studies are needed to compare the efficacy and cosmetic outcomes of different approaches for different degrees of epicanthal folds. There is also a need for further well‐designed studies evaluating the efficacy and clinical impact of different postoperative scar modification approaches.

## Author Contributions

Conceptualization: F.A.F., R.C.A., A.Z.K. Methodology: F.A.F., A.Z.K. Investigation: F.A.F., R.C.A. Writing – original draft: F.A.F., A.K. Writing – review and editing: F.A.F., R.C.A., E.B., K.A.T., T.P.U., A.Z.K. Supervision: K.A.T., T.P.U., A.Z.K. Project administration: E.B., A.K.

## Funding

The authors have nothing to report.

## Disclosure

The authors have nothing to report.

## Conflicts of Interest

The authors declare no conflicts of interest.

## Supporting information


**Table S1:** Studies evaluating scarring following medial epicanthoplasty.

## Data Availability

Data sharing not applicable to this article as no datasets were generated or analysed during the current study.
