# Supplementary material for: Minimizing Postoperative Scars in Epicanthoplasty: A Concise Review
Source: J Cosmet Dermatol. 2025 Dec 10;24(12):e70603. doi: 10.1111/jocd.70603 (PMC12690353; doi:10.1111/jocd.70603)
Supplement: Supplementary file 1 — Table S1: Studies evaluating scarring following medial epicanthoplasty. [file JOCD-24-e70603-s001.docx]

Supplementary Table 1. Studies evaluating scarring following medial epicanthoplasty.

| Author/Year/Reference | Number of participants (n)/ mean age (years) | Summary |
| --- | --- | --- |
| Redraping | | |
| Oh/2007/[1] | 215/22.5 | Tension-free skin redraping method. Excision of OOM underlying the epicanthal fold and trimming of skin flap. No flap design, plication or subdermal fixation. No hypertrophic scarring. |
| Kim/2018/[2] | 525/20.2 | Boomerang epicanthoplasty, a modified skin redraping approach using skin excision resulting in a boomerang shaped defect. No excision or release of OOM. Average patient satisfaction was 4.6 (1-5, 5=best). |
| Sun/2019/[3] | 475/26 | Double eyelid plasty combined with a modified skin redraping technique with excision of preseptal and a small amount of pretarsal OOM along the supratarsal incision. No cases of hypertrophic scarring were reported, 97% of patients were satisfied. |
| Mo/2021/[4] | 147/26.5 | Modified skin redraping method with horizontal point incision, staged “Y-shaped” dog ear correction and release of dense connective tissue between the dermis and the OOM. 98.6% reported minimal or nonvisible scarring. |
| Subcutaneous epicanthoplasty | | |
| Jordan/1989/[5] | 10/5-18 | Skin incised along the epicanthal fold, underlying OOM removed, skin edges sutured to the periosteum. One case of scarring. |
| Lee/2000/[6] | 67/15-45 | Augmentation rhinoplasty followed by double eyelidplasty concluded with anchor epicanthoplasty. Removal of the preseptal portion of OOM and superficial fibres of the MCL. Anchoring of medial portion of incision from double eyelidplasty to MCL or periosteum of nasal bone. No hypertrophic scarring reported. |
| Yen/2002/[7] | 38/n.d. | No-scar epicanthoplasty with concomitant upper blepharoplasty or aponeurotic ptosis surgery. Elevation of dermis on the medial aspect of blepharoplasty incision with skin hooks and consequent dissection and excision of OOM. Could be combined with deep tissue fixation of the medial edge of the blepharoplasty incision for greater elimination of epicanthal fold. The authors report no scarring, however, “some cases” of under-correction were noted. |
| Ni/2016/[8] | 118/23 | Scarless epicanthoplasty with concomitant upper blepharoplasty involving subcutaneous dissection through medial end of upper blepharoplasty incision, OOM cut, adhesion bands trimmed before anchoring of OOM to new subdermal locations. No scarring in the medial canthal region. Patient satisfaction rated 1-5 (5=best): grade 1: 2; grade 2: 5; grade 3: 17; grade 4: 83; grade 5: 11. |
| Esmaeilkhanian/2021/[9] | 89/31.6 | Anchor epicanthoplasty with removal of aponeurotic fat, preseptal OOM and superficial fibres of the MCL. Anchoring of medial skin to deeper tissue. Mean MSSS 5.4. Histopathological comparisons of epicanthal folds revealed no difference between Asian and non-Asian groups. |
| Y-V epicanthoplasty | | |
| Kao/1998/[10] | 148/27.3 | Modified Y-V advancement procedure: Excision of triangular piece of dermis, removal of excessive OOM, closure with two-layer method. One case of hypertrophic scarring treated with local steroid injections. |
| Lee/2006/[11] | 118/25.2 | The authors describe a periciliary Y-V epicanthoplasty where a) every skin incision should be close to the skin-mucosal junctions and eyelashes to diminish visible scars, b) great care should be taken when undermining subcutaneously around the lacrimal puncta and canaliculi, not too deep to the OOM, c) over-resection of the redundant skin is avoided when trimming the lower triangular flap. No hypertrophic scarring reported. |
| Li/2008/[12] | 92/23.2 | Y-V advancement including trimming of excessive OOM, release of all adhesion bands and anchoring to periosteum of nasal bone or MCL. Three cases of hypertrophic scarring regressing without intervention. |
| Zhao/2010/[13] | 68/26.8 | Modified Y-V plasty with incision lines along eyelashes and skin-mucosal junctions, release of OOM, flap anchoring to periosteum. Early, transient scar proliferation noted fading by 3 months. No hypertrophic scarring reported. |
| Z-epicanthoplasty | | |
| Lessa/1984/[14] | 31/ n.d. | New canthi marked as in the quadrangular flap approach by Mustardé followed by double Z-plasty. Excision of fat and fibrous tissue. Nasal transfixation of MCL. One case of hypertrophic scarring and 4 cases of visible scarring. |
| Park/1996/[15] | 160/n.d. | Z-epicanthoplasty, underlying OOM included in excision, dermis anchored to the deeper tissues. Three patients had persistent redness from the anchoring sutures requiring removal. Three patients developed scarring; one underwent revisional surgery. |
| Park/2000/[16] | 297/n.d. | Modified Z-epicanthoplasty including excision of fibrofatty tissue and OOM, may include anchoring of dermis to deeper tissues. Three patients developed anchoring suture granulomas. Three cases of visible scarring, two requiring revision, one treated with steroid injections. |
| Yoo/2002/[17] | 30/25 | Root Z-epicanthoplasty. Release of muscle and fibrofatty tissue underlying the epicanthal fold, no skin excision. No scarring requiring intervention. |
| Zhang/2006/[18] | 67/17-45 | Concomitant double eyelid plasty and modified Z-plasty with partial excision of OOM and MCL and plication of MCL. Mild scarring present in all patients, gradually fading, but remaining visible. No hypertrophic scarring reported. |
| Liu/2011/[19] | 163/23 | Double eyelid plasty followed by modified Z-plasty, OOM partially attached to one flap during transposition. Authors report no visible scarring. |
| Lu/2011/[20] | 322/25.6 | Modified Z-plasty with double eyelid plasty, excision of pretarsal connective tissue and OOM, release of preseptal OOM, periosteal anchoring. Three cases of hypertrophic scarring that gradually faded without intervention. |
| Hu/2012/[21] | 23/23.6 | Double Z-plasty with release of canthal tension by OOM dissection. No hypertrophic scarring reported. |
| Lai/2012/[22] | 86/29.5 | Modified Z-plasty with resection of pretarsal and preseptal portions of OOM and plication of the MCL when longer than 5 mm. Hypertrophic scarring developed in 2 patients which responded to intralesional steroid injections. |
| Wang/2013/[23] | 873/22 | Modified Z-plasty with upper blepharoplasty. Complete release of dermis, incision through unspecified ligament underlying skin flap and flap rotation. No hypertrophic scarring reported. |
| Lyu/2016/[24] | 48/24.6 | Stepwise modified Z-plasty, excision of fibrous tissue and OOM between the dermis and MCL. Scarring graded 0-3 (0=best): grade 0: 10.4%; grade 1: 72.9%; grade 2: 16.7%; grade 3: 0%. |
| Zhao/2016/[25] | 112/23 | Upper blepharoplasty and modified Z-plasty combination with excision of OOM, tarsal fat and fibrous tissue. An undisclosed number of patients developed transient hypertrophic scarring reportedly fading within 12 months. |
| Wu/2018/[26] | 241/27 | Modified Z-plasty with double eyelid plasty. Eyelid plasty performed first to avoid impact of tension change from eyelid plasty impacting epicanthoplasty. Tension free closure due to determination of one triangular flap by the other. Avoidance of linear scar contracture by using discontinuous sutures. Partial excision of OOM and orbital adipose tissue. |
| Zhao/2018/[27] | 132/24 | Upper blepharoplasty and modified Z-plasty combination with excision of OOM, tarsal fat and fibrous tissue. Patient reported outcome measure questionnaires regarding scarring of the inner canthus graded 0-3 (3=best) revealed: grade 0: 9.1%; grade 1: 10.6%; grade 2: 42.4%; grade 3: 37.9%. |
| Huang/2019/[28] | 30/23.6 | Z-plasty with release of flap from medial canthal tendon and partial excision of OOM. Split face randomization to injections with saline or botulinum toxin type A injections, with better VSS scores in the latter group. |
| Lin/2019/[29] | 104/21.2 | Stepwise, integrated Z-plasty with anchoring of the skin-muscle flap to the MCL and transposition of the OOM. All scars regarded as invisible or only visible under close inspection. |
| Suo/2020/[30] | 285/24 | Modified Z-plasty with or without double eyelid plasty. Excision of OOM, underlying fat and preaponeurotic fat pads according to need. Subcutaneous detachment and repositioning of OOM in the MCL prior to flap rotation. 91% of patients were satisfied with the scar, 9% complained of too visible scarring. Four cases of revision performed due to excessive scar formation. |
| Aung/2021/[31] | 72/24 | 3-dimensional Z-epicanthoplasty followed by double eyelid plasty. Release of tension and plication of the MCL with anchoring to the deeper tissues. 40% of patients had no scarring, 58% had minimal scarring and 1% had more visible scarring, none had severe scarring. |
| Tong/2021/[32] | 117/24,3 | Modified Z-epicanthoplasty following double-eyelid blepharoplasty with excision of preseptal fibrous tissue and OOM. 83.3% had no visible scarring, 15.4% had minimal scarring visible only under close inspection, 1.3% had visible scarring, 1 patient had severe scarring requiring revision. |
| Li/2022/[33] | 135/26 | Z-plasty consisting of dual-plane epicanthoplasty. Creation of first flap and excision of OOM followed by upper blepharoplasty before creation of lower epicanthoplasty flap. 85% of patients reported minimum or barely visible scarring. 3% reported hypertrophic scarring. |
| Su/2022/[34] | 138/23.4 | 3-dimensional Z-plasty rotating and turning inward the excess skin from the horizontal direction to a final sagittal orientation supplementing the medial canthal skin deficiency, OOM released from dermis. No hypertrophic scarring reported. |
| Wijaya/2022/[35] | 489/24.5 | Thunderbolt Z-plasty with excision of fibrous tissue and hypertrophied OOM as well as plication of the medial canthal ligament. 1.2% developed cicatricial redness gradually fading during follow-up, no hypertrophic scarring reported. |
| Fatani/2023/[36] | 35/34 | Modified Z-plasty with release of flap from underlying medial canthal ligament in patients with Fitzpatrick skin type III-V. Median scar visibility was 3/10 with 83% of patients with no scar elevation or hypertrophy. No difference across Fitzpatrick skin type was found. |
| Comparative studies | | |
| Park/2013/[37] | 99/24 | Ptosis correction combined with epicanthoplasty, using either elliptical excision epicanthoplasty, half-Z epicanthoplasty, periciliary epicanthoplasty or V-W epicanthoplasty. Seven patients complained of scarring after epicanthoplasty, all cases resolved without intervention. No cases of hypertrophic scarring reported. |
| Wang/2013/[38] | 252/25.3 | Comparison of horizontal incision method, Z-plasty and V-W plasty, approach chosen in this order depending on epicanthal fold severity. All approaches included excision of underlying fibrous tissue and OOM. Hypertrophic scarring necessitating steroid injections were reported in 12 cases. Unfortunately, the study does not report differences in scarring tendency between the surgical approaches. |
| Kim/2015/[39] | 46/11 | Comparing the surgical outcome of Z-plasty with release of fibrous tissue and OOM; Y-V plasty with release of OOM; Mustardé four flap technique or the one-armed jumping man modification with periosteal anchoring. Z-plasty resulted in least scarring, followed by Y-V plasty. The Mustardé technique gave the most scarring but was also used in the most severe cases. |
| Liu/2017/[40] | 47/group 1: 21.3; group 2: 20.7 | Comparing superior double eyelid plasty with medial epicanthoplasty without (group 1) or with (group 2) resection of OOM and plication of the MCL. Group 2 had a greater reduction in inner intercanthal distance as well as less of a tendency for hypertrophic scarring. |
| Zeng/2017/[41] | 30/24.3 | Patients underwent double eyelid plasty and Z-epicanthoplasty. For the Z-epicanthoplasty. an epicanthal tension-releasing incision based on the skin projection of the inner canthal ligament was found to be more effective and safer than an incision parallel to the lower inner canthal mucocutaneous junction. No hypertrophic scarring reported. |
| Wang/2018/[42] | 106/25 | Comparison of modified Z-plasty and modified skin redraping methods. Release of dermis, no excision of OOM. The approaches had similar patient satisfaction scores. Z-plasty caused significantly more scarring. |
| Ding/2021/[43] | 43/26 | Comparison of a modified Z-plasty and a modified Y-V flap approach to correct congenital epicanthus. Three blinded, independent specialists evaluated scars with VSS and concluded that Y-V flap method resulted in significantly less scarring while the Z-plasty technique was more proficient in shortening the ICD. |
| Wang/2024/[44] | 104/28 | V-Y flap with (n=83) or without (n=21) OOM included in the skin flap for myocutaneous ring reconstruction. The former group achieved higher patient satisfaction, including subjective patient scar evaluation. |
| He/2025/[45] | 172/six groups, around 28 | Modified asymmetric inverse Z-plasty (n=94) vs Z-plasty (n=78). Higher patient satisfaction and lower VSS scores in patients treated with modified asymmetric inverse Z-plasty. |
| Kim/2025/[46] | 199/25 | Comparison of the elliptical excision method (n=94) and the mini-redraping method (n=105). In the elliptical excision group 12 patients reported under correction and two patients had prominent scarring In the mini-redraping group 4 patients reported under correction and none reported excessive scarring. |
| Liu/2025/[47] | 20/29 | Patients underwent bilateral modified transverse incision technique epicanthoplasty with release of OOM. Postoperatively one eye was randomized to receive botulinum toxin injection and the other saline injection. At six months, treated eyes had better modified Stony Brook Scar Evaluation Scale score, Patient Scar Assessment Scale score, VAS score and average scar width. |
| Other | | |
| del Campo/1984/[48] | 49/n.d. | Creation of a transposition flap based in the medial canthal region taken from the posterior surface of the epicanthal fold placed over the lacrimal crest. Plication of MCL. Anchoring of the MCL in cases of posttraumatic telecanthus. One case of hypertrophic scarring reported. |
| Yoon/1993/[49] | 4/6-26 | Modified Mustardé technique “one-armed jumping man incision” resulting in less scarring. |
| Lin/2000/[50] | 85/n.d. | VM-plasty consisting of single V-flap from the inner surface and a double V-flap from the outer surface of the epicanthal fold. Performed in conjunction with double eyelid plasty. Release of, but not excision of OOM. Redness of scar was a common complaint which usually faded. No report of unsightly scarring. |
| Cho/2002/[51] | 10/27.3 | Y-W-plasty or inverted Y-V plasty with plication of the MCL and periosteal anchoring of the dermis. No hypertrophic scarring reported. |
| Yi/2007/[52] | 52/26 | Simple epicanthoplasty with minimal scar (SEMS), OOM released over the medial canthus. Periosteal fixation of flap to nasal bone. No hypertrophic scarring reported. Two patients developed depressed scars in need of correction. |
| Chen/2009/[53] | 82/26 | Periciliary approach with removal of connective tissue, muscular fibres, and the supraciliary portion of OOM around the MCL. Followed by double eyelid plasty in most cases. No visible scarring following epicanthoplasty. |
| Liu/2012/[54] | 62/24 | Inverted V-Y advancement with vertical V-shaped skin flap, individualized excision of OOM, most often combined with double eyelid plasty. Transient swelling and tenderness, no complaints about scarring. |
| Liu/2012/[55] | 156/26 | Lazy S-curve epicanthoplasty with curvilinear skin incision, removal of OOM around MCL, anchoring to periosteum. No hypertrophic scarring reported. |
| Park/2014/[56] | 60/24 | Epicanthoplasty performed as modified elliptical excision method, with additional incision parallel with the ciliary margin to avoid formation of vertical scar nasally. OOM not released. One case of hypertrophic scarring in need of local steroid injections. |
| Seo/2014/[57] | 1132/26.2 | A tension-free “inside-out” technique in which a triangular flap is moved anteriorly. Release of OOM. Nine patients complained of visible scarring after 2 months. |
| Zan/2016/[58] | 118/25.5 | Double eyelid plasty followed by U-flap epicanthoplasty with excision of fibrous tissue and OOM. Fixation of flap to medial portion of the medial canthal ligament or nasal periosteum. 97.5% patient satisfaction. Three patients developed hypertrophic scarring requiring local steroid injections. |
| Jin/2017/[59] | 136/25.2 | The authors describe a tension-free epicantoplasty technique that is performed in conjunction with double eyelid surgery where the incisions are concealed along the subciliary lines, the medial canthus is reconstructed, and fibrous tissue and excessive OOM superior to the MCL is removed. Scarring graded 0-3 (0=best): grade 0: 88.2%; grade 1: 10.3%; grade 2: 1.5%; grade 3: 0%. |
| Zhang/2018/[60] | 423/29.8 | V-flap epicanthoplasty with partial resection of OOM and MCL plication. Postoperative steroid injection. 82.7% patient satisfaction. 17.3% developed hypertrophic scarring, 9.22% of which remained visible at 6 months despite local steroid injections. |
| Zhang/2018/[61] | 753/27.8 | Modified epicanthoplasty with upper arch flap and extended lower eyelid incision. OOM crossing MCL was cut and partially resected with fibrous tissue followed by MCL plication. 90% of patients “very satisfied”, no hypertrophic scarring reported. |
| Mao/2019/[62] | 216/27.9 | Dual-plane epicanthoplasty, usually with concomitant double eyelid plasty. Procedure involves creation of an advanced skin flap and a rotation skin flap. Release of connective tissue and OOM. Wound erythema common first two months. Hypertrophic scarring reported in one eye. Rest of cases reported with inconspicuous scarring. |
| Xie/2019/[63] | 236/23.6 | Double eyelid plasty followed by combined transverse and pouch incisions epicanthoplasty. Transverse incision in the new inner canthus followed by incision along lower eyelid. Excision of OOM. Scarring graded 0-3 (0=best): grade 0: 15.3%; grade 1: 70.3%; grade 2: 14.4%; grade 3: 0%. |
| Cao/2021/[64] | 40/33.6 | A modified rectangular flap method in conjunction with double eyelid plasty. Release of flap from underlying tendon without excision to facilitate tension-free redraping. Scarring was graded 0-3 (0=best) with 12.5% graded 0, 85% graded 1, 2.5% graded 2 and 0% graded 3. |
| Park/2021/[65] | 712/23.7 | The authors describe a 45-degree upward tension-releasing technique performed with or without double eyelid plasty. Tension was released in the medial epicanthal area by separation of the dense connective tissue between the dermis and OOM. Six patients developed hypertrophic scarring necessitating steroid injections. 95% of patients were satisfied. |
| Yang/2021/[66] | 547/25 | Lower margin palpebral incision epicanthoplasty with release of OOM, incision and refixation of MCL. Double eyelid plasty after epicanthoplasty. 98% patient satisfaction. Authors report tiny scars in the medial canthal area. 2% complained of scarring and 5 cases required revision surgery. |
| Yang/2021/[67] | 118/24.7 | Rotated-advanced-back cut flap epicanthoplasty, consisting of triangular flap crossing the lacrimal lake with release of dermis from MCL. 73% of patients rated the results as perfect, 27% as good, none rated as dissatisfied or failed. |
| Kim/2023/[68] | 421/28 | Proposing that excessive undermining is scar-provoking, the authors describe a technique with minimal undermining consisting of a triangular skin resection, release of OOM, release of upper half of MCL, and dog ear correction. The authors reported that this technique resulted in zero cases of hypertrophic scarring. |
| Park/2024/[69] | 18/31.3 | Y-W epicanthoplasty with resection of subcutaneous and muscle tissue superficial to the MCL and release of adhesion band under the epicanthal fold. 11 patients had no apparent scar; 6 patients had minimal scarring only visible at close inspection; and 1 patient developed hypertrophic scarring treated with triamcinolone injections. |
| Chen/2025/[70] | 306/n.d. | Five-step medial epicanthoplasty with release of dense connective tissue, resection of OOM and plication of MCL. Hypertrophic scarring in 85 patients successfully treated with silicone gel, triamcinolone injections and laser therapy. |
| Liu/2025/[71] | 114/26 | Skin replacement epicanthoplasty and concomitant septoaponeurosis junctional thickening double eyelidplasty. Release of OOM and plication of MCL. Average VSS was 1.1, no hypertrophic scarring reported. |
| Long/2025/[72] | 100/30 | Modified inverted ‘L’ epicanthoplasty combined with upper blepharoplasty. Release of OOM and plication of MCL. One case of scarring that resolved within 3 months. |
| Wang/2025/[73] | 40/25 | Medial canthal fibrous band epicanthoplasty with upper blepharoplasty. Severing of OOM and resection of medial canthal fibrous tissue. OSAS and POSAS improving from one to three months, numerical values not disclosed. |
| Correction of congenital anomalies | | |
| Jung/2011/[74] | 17/5.7 | Correction of epiblepharon with tension free redraping method including release of dense connective tissue and resection of OOM. No hypertrophic scarring, high cosmetic satisfaction. |
| Sa/2012/[75] | 16/3.6 | Patients with BPES. Skin redraping method including excision of fibrous tissue and OOM, plication of the MCL and trimming of fibroadipose tissue under the nasal skin. Scarring graded 0-3 (0=best). Grade 0: 18.8%; grade 1: 68.8%; grade 2: 12.5%; grade 3: 0%. |
| Oh/2014/[76] | 12/7.5 | Epiblepharon correction only through skin redraping epicanthoplasty. Plication of MCL and subcutaneous tissue as well as partial excision of OOM. Cilia touch was corrected in all patients. One patient developed mild scarring that resolved without intervention. |
| Ni/2017/[77] | 38/7.6 (only group 2 relevant here) | Modified Hotz procedure with modified Z-plasty for epiblepharon. Partial excision of OOM. Addition of epicanthoplasty improved results of Hotz procedure. VSS of the medial canthus was 1.13. |
| Chen/2019/[78] | 78/6.6 | Modified Z-flap referred to as half Z epicanthoplasty as part of correcting congenital epiblepharon with epicanthus. Dissection, release and partial excision of OOM. No discernible scarring reported. |
| Choi/2019/[79] | 12/1-33 | Correction of congenital telecanthus with tension free skin redraping, limited resection of OOM. All scars reported as barely visible. |
| Hu/2021/[80] | 18/6.2 | Correction of recurrent epiblepharon with skin redraping approach with excision of OOM. Report almost no visible scarring. |
| Mimura/2022/[81] | 25/11.4 | Correction of epiblepharon with combined Hotz procedure and rotational flap epicanthoplasty including excision of pretarsal OOM. No unfavourable scarring reported. |
| Hu/2023/[82] | 53/5.3 | Minimal lower eyelid epicanthoplasty for correction of epiblepharon. Infraciliary incision with excision of superficial OOM. No obvious scarring reported. |
| Ma/2023/[83] | 9/23 | Correction of congenital entropion with skin redraping and modified Hotz procedure. No hypertrophic scarring reported. |
| Medel/2024/[84] | 22/2.9 | Lambda-double-fixation medial epicanthoplasty correcting BPES. Lambda shaped incision, removal of fibroadipose tissues surrounding MCL which was then cut and excised with subsequent periosteal anchoring. |
| Watanabe/2025/[85] | 28/7.6 | Modified Hotz procedure with or without Y-V epicanthoplasty for epiblepharon. Lower rate of recurrence in patients with severe epicanthal folds who underwent both procedures. No hypertrophic or significant scarring reported. |

BPES: blepharophimosis-ptosis-epicanthus inversus syndrome; ICD: intercanthal distance; MCL: medial canthal ligament; MSSS: Manchester scar scale score (5-28); n.d.: not described; OOM: orbicularis oculi muscle; OSAS: Observer Scar Assessment Scale; POSAS: Patient and Observer Scar Assessment Scale; VAS: visual analogue scale; VSS: Vancouver scar scale

References:

[1] Oh YW, Seul CH, Yoo WM. Medial epicanthoplasty using the skin redraping method. Plast Reconstr Surg. 2007;119:703-10.

[2] Kim JH, Hwang K, Park B. The Boomerang Epicanthoplasty: A Technique That Avoids Hooding. J Craniofac Surg. 2018;29:1813-6.

[3] Sun W, Yin N, Song T, Wu D, Li H, Wang Y. A practical technique combining orbicularis oculi muscle resection-based epicanthoplasty and orbicularis-tarsus fixation double-eyelid plasty for cosmetic blepharoplasty. J Plast Reconstr Aesthet Surg. 2019;72:2009-16.

[4] Mo YW, Jung GY. Surgical Results and Patient Satisfaction After A New Surgical Technique for Asian Medial Epicanthoplasty: A Modified Skin Redraping Method Using a Horizontal Point Incision and Staged 'Y-Shaped' Dog Ear Correction. Ann Plast Surg. 2021;87:389-95.

[5] Jordan DR, Anderson RL. Epicanthal folds. A deep tissue approach. Arch Ophthalmol. 1989;107:1532-5.

[6] Lee Y, Lee E, Park WJ. Anchor epicanthoplasty combined with out-fold type double eyelidplasty for Asians: do we have to make an additional scar to correct the Asian epicanthal fold? Plast Reconstr Surg. 2000;105:1872-80.

[7] Yen MT, Jordan DR, Anderson RL. No-scar Asian epicanthoplasty: a subcutaneous approach. Ophthalmic Plast Reconstr Surg. 2002;18:40-4.

[8] Ni F, Luo S, Yu D, Zhu Y, Shang Y, Chen Y, et al. Scarless Epicanthoplasty and Concomitant Double Eyelidplasty in Chinese Eyelids. Aesthetic Plast Surg. 2016;40:840-5.

[9] Esmaeilkhanian H, Kashkouli MB, Abdolalizadeh P, Aghamirsalim M, Shayanfar N, Karimi N. Revisiting Anchor Epicanthoplasty in Mild to Moderate Asian Epicanthal Folds: A Clinicopathological Study. Aesthetic Plast Surg. 2021;45:181-90.

[10] Kao YS, Lin CH, Fang RH. Epicanthoplasty with modified Y-V advancement procedure. Plast Reconstr Surg. 1998;102:1835-41.

[11] Lee YJ, Baek RM, Song YT, Chung WJ, Lee JH. Periciliary Y-V epicanthoplasty. Ann Plast Surg. 2006;56:274-8.

[12] Li FC, Ma LH. Double eyelid blepharoplasty incorporating epicanthoplasty using Y-V advancement procedure. J Plast Reconstr Aesthet Surg. 2008;61:901-5.

[13] Zhao YQ, Luo DA. Modified Y-V epicanthoplasty with raised medial canthus in the Asian eyelid. Arch Facial Plast Surg. 2010;12:274-6.

[14] Lessa S, Sebastia R. Z-epicanthoplasty. Aesthetic Plast Surg. 1984;8:159-63.

[15] Park JI. Z-epicanthoplasty in Asian eyelids. Plast Reconstr Surg. 1996;98:602-9.

[16] Park JI. Modified Z-epicanthoplasty in the Asian eyelid. Arch Facial Plast Surg. 2000;2:43-7.

[17] Yoo WM, Park SH, Kwag DR. Root z-epicanthoplasty in asian eyelids. Plast Reconstr Surg. 2002;109:2067-71; discussion 72-3.

[18] Zhang H, Zhuang H, Yu H, Feng Y, Wang T, Hu S, et al. A new Z-epicanthoplasty and a concomitant double eyelidplasty in Chinese eyelids. Plast Reconstr Surg. 2006;118:900-7.

[19] Liu Y, Huang J, Wen K. A modified and accurately designed Z-epicanthoplastic technique. Aesthetic Plast Surg. 2011;35:1112-6.

[20] Lu JJ, Yang K, Jin XL, Xu JJ, Zhang C, Zhang B, et al. Epicanthoplasty with double eyelidplasty incorporating modified Z-plasty for Chinese patients. J Plast Reconstr Aesthet Surg. 2011;64:462-6.

[21] Hu X, Lin X, Ma G, Jin Y, Chen H, Chen X, et al. Two-Z-epicanthoplasty in a three-dimensional model of Asian eyelids. Aesthetic Plast Surg. 2012;36:788-94.

[22] Lai CS, Lai CH, Wu YC, Chang KP, Lee SS, Lin SD. Medial epicanthoplasty based on anatomic variations. J Plast Reconstr Aesthet Surg. 2012;65:1182-7.

[23] Wang L, Chen X, Zheng Y. A modified z-epicanthoplasty combined with blepharoplasty used to create an in-type palpebral fissure in Asian eyelids. Aesthetic Plast Surg. 2013;37:704-8.

[24] Lyu D, Jin Y, Chang L, Chen H, Ma G, Yu W, et al. The Modified Z-Epicanthoplasty-A Stepwise and Individualized Design. Ann Plast Surg. 2017;78:7-11.

[25] Zhao J, Qi Z, Zong X, Yang X, Song G, Du L, et al. A Modified Method Combining Z-Epicanthoplasty and Blepharoplasty to Develop Out-Fold Type Double Eyelids. Aesthetic Plast Surg. 2016;40:48-53.

[26] Wu S, Guo K, Xiao P, Sun J. Modifications of Z-Epicanthoplasty Combined with Double-Eyelid Blepharoplasty in Asians. Aesthetic Plast Surg. 2018;42:226-33.

[27] Zhao JY, Guo XS, Song GD, Zong XL, Yang XN, Du L, et al. Surgical outcome and patient satisfaction after Z-epicanthoplasty and blepharoplasty. Int J Ophthalmol. 2018;11:1922-5.

[28] Huang RL, Ho CK, Tremp M, Xie Y, Li Q, Zan T. Early Postoperative Application of Botulinum Toxin Type A Prevents Hypertrophic Scarring after Epicanthoplasty: A Split-Face, Double-Blind, Randomized Trial. Plast Reconstr Surg. 2019;144:835-44.

[29] Lin Y, Chen B, Woo DM, Lee JKS, Liu J, Zhu X, et al. Integrated and Stepwise Epicanthoplasty Combined with Blepharoplasty (ISEB) in an Ethnic Chinese Population. Aesthetic Plast Surg. 2019;43:1235-40.

[30] Suo L, Li J, Fu R, Xie Y, Huang RL. A Four-Step Technique for Creating Individual Double-Eyelid Crease Shapes: A Free-Style Design. Plast Reconstr Surg. 2020;146:756-65.

[31] Aung ZM, Yang X, Chen X, Jun MW, Zhang Y, Han D, et al. Experience of Comprehensive Three Dimensional Epicanthoplasty With Upper Blepharoplasty in Asian Patients. Ann Plast Surg. 2021;86:S235-S8.

[32] Tong J, Wang R, Sun J, Yang J. Modified Z-Epicanthoplasty Based on the Movement and Tension of Medial Canthal Area in Asian Females. J Craniofac Surg. 2021;32:2198-201.

[33] Li X, Wang J, Ma J, Yu L, Wang T. Asian upper blepharoplasty and dual-plane epicanthoplasty: An integrated four-step technique. J Cosmet Dermatol. 2022;21:1111-9.

[34] Su Z, Liu L, Fan J, Tian J, Gan C, Jiao H, et al. Three-Dimensional Z-Epicanthoplasty Retains the Depth of the Medial Canthus With Scars That Are Less Visible. Ann Plast Surg. 2022;89:17-22.

[35] Wijaya WA, Tang J, Zhong A, Zhou K, Liu Y, Yang A, et al. A Novel Thunderbolt Z-Epicanthoplasty for Asians. Aesthetic Plast Surg. 2022;46:2833-40.

[36] Fatani DR, Alsuhaibani OS, Alsuhaibani AH. Cosmetic outcomes of epicanthoplasty for epicanthus tarsalis. Saudi J Ophthalmol. 2023;37:94-9.

[37] Park DH, Park SU, Ji SY, Baik BS. Combined epicanthoplasty and blepharoptosis correction in Asian patients. Plast Reconstr Surg. 2013;132:510e-9e.

[38] Wang S, Shi F, Luo X, Liu F, Zhou X, Yang J, et al. Epicanthal fold correction: our experience and comparison among three kinds of epicanthoplasties. J Plast Reconstr Aesthet Surg. 2013;66:682-7.

[39] Kim CY, Lee SY. Structural and cosmetic outcomes of medial epicanthoplasty: An outcome study of three different techniques. J Plast Reconstr Aesthet Surg. 2015;68:1346-51.

[40] Liu HP, Zhao YY, Li B, Qi J, Zhang D. The orbicularis oculi muscle resection technique for medial epicanthoplasty: A retrospective review of surgical outcomes in 47 Chinese patients. J Plast Reconstr Aesthet Surg. 2017;70:96-103.

[41] Zeng L, Cen Y, Chen J, Lei L. Epicanthoplasty with Epicanthal Dermatic Tension-Releasing Incision Based on Skin Projection of Inner Canthal Ligament. Aesthetic Plast Surg. 2017;41:863-71.

[42] Wang G, Zhang S, Ma J, Li D, Xue H. Cosmetic and structural outcomes of two different techniques of medial epicanthoplasty according to epicanthal fold classification and severity: A cohort study. J Plast Reconstr Aesthet Surg. 2018;71:1453-61.

[43] Ding F, Zhao ZF, Lu L, Liu F, Sun D, Luo X, et al. Experience of Two Different Techniques of Medial Epicanthoplasty with Four-Point Homologous Design: A Cohort Study. Aesthetic Plast Surg. 2021;45:1581-90.

[44] Wang B, Zhang S, Chen Y, Liu Z, Yu J, Zhou H, et al. A comparative retrospective analysis: myocutaneous flap versus skin flap in V-Y medial epicanthal fold reconstruction. Front Surg. 2024;11:1335796.

[45] He Z, Zhang W, Yu X, Qin H, Teng J, Xie J. Comparison of Modified Asymmetric Inverse Z-plasty and Z-plasty in the Correction of Epicanthal Folds. Aesthetic Plast Surg. 2025.

[46] Kim JH, Kim H. Simple Approach to Cosmetic Medial Epicanthoplasty: A Modification of the Skin Redraping Method. Ann Plast Surg. 2025;94:145-51.

[47] Liu D, Ou Y, Luo L, Wu M, Zhang Y, Chen L, et al. The Efficacy and Safety of Botulinum Toxin Type A in Prevention of Hypertrophic Scars After Epicanthoplasty: A Split-Face Double-Blinded Randomized Controlled Trial. Aesthetic Plast Surg. 2025;49:1227-38.

[48] del Campo AF. Surgical treatment of the epicanthal fold. Plast Reconstr Surg. 1984;73:566-71.

[49] Yoon K. Modification of Mustarde technique for correction of epicanthus in Asian patients. Plast Reconstr Surg. 1993;92:1182-6.

[50] Lin SD. Correction of the epicanthal fold using the VM-plasty. Br J Plast Surg. 2000;53:95-9.

[51] Cho BC, Lee KY. Medial epicanthoplasty combined with plication of the medial canthal tendon in Asian eyelids. Plast Reconstr Surg. 2002;110:293-300; discussion 1.

[52] Yi SK, Paik HW, Lee PK, Oh DY, Rhie JW, Ahn ST. Simple epicanthoplasty with minimal scar. Aesthetic Plast Surg. 2007;31:350-3.

[53] Chen W, Li S, Li Y, Wang Y. Medial epicanthoplasty using the palpebral margin incision method. J Plast Reconstr Aesthet Surg. 2009;62:1621-6.

[54] Liu L, Li S, Fan J, Gan C, Tian J, Jiao H, et al. Inverted 'V--Y' advancement medial epicanthoplasty. J Plast Reconstr Aesthet Surg. 2012;65:43-7.

[55] Liu Y, Lei M, Wang Y, Mu X. Lazy S-curve epicanthoplasty in Asian blepharoplasty. Aesthetic Plast Surg. 2012;36:254-60.

[56] Park DH, Park SU, Lee BK, Lee YB, Do ER, Han DG, et al. Medial epicanthoplasty without a vertical scar. Ann Plast Surg. 2014;73:8-11.

[57] Seo JD, Kim JH, Pak CS, Heo CY. Medial epicanthoplasty using the "inside-out" technique. J Plast Surg Hand Surg. 2014;48:139-42.

[58] Zan T, Jin R, Li H, Herrler T, Meng X, Huang X, et al. A Novel U-Flap Epicanthoplasty for Asian Patients. Aesthetic Plast Surg. 2016;40:458-65.

[59] Jin Y, Lyu D, Chen H, Ma G, Qiu Y, Zou Y, et al. Invisible scar medial epicanthoplasty: A novel approach. J Plast Reconstr Aesthet Surg. 2017;70:952-8.

[60] Zhang S, Xue HY. Adjustable V-Flap Epicanthoplasty Based on Desired Eyelid Morphology. Aesthetic Plast Surg. 2018;42:1571-5.

[61] Zhang S, Xue H. Upper Arch Flap Combined with Extended Incision of Lower Eyelid: A Modified Epicanthoplasty in Correcting Epicanthus. Aesthetic Plast Surg. 2018;42:1033-8.

[62] Mao R, Zhou L, Yu L, Wang T, Wang J. Dual-plane epicanthoplasty in Chinese blepharoplasty. J Cosmet Dermatol. 2020;19:3323-30.

[63] Xie A, Cao Y, Yu D. Combined Transverse Incision and Pouch Incision for the Correction of Medial Epicanthus. J Craniofac Surg. 2019;30:1499-502.

[64] Cao Z, Guo YQ, Tan SX, Niu CY, Wang JH, Miao CL, et al. The Modified Rectangle Flap Epicanthoplasty: A Novel and Individualized Design. Aesthetic Plast Surg. 2021;45:564-9.

[65] Park SE, Choi BK, Roh TS, Lew DH, Jung BK. A 45-degree Upward Tension-Releasing Epicanthoplasty Performed in 712 Consecutive Cases. J Plast Reconstr Aesthet Surg. 2021;74:3101-7.

[66] Yang F, Zhang J, Gu C, Chen W. Medial Epicanthoplasty Using a Lower Palpebral Margin Incision Combined With a Tiny Triangular Flap. Aesthetic Plast Surg. 2021;45:1056-63.

[67] Yang L, Huang Y, Li H. Epicanthoplasty With Rotated-advanced-back Cut Flap. J Craniofac Surg. 2021;32:1526-8.

[68] Kim T, Lee JH, Kim H, Jun D. Triangular Resection Epicanthoplasty: A Novel Method of Minimizing Hypertrophic Scar After Medial Epicanthoplasty. J Craniofac Surg. 2023;34:1340-2.

[69] Park H, Moon Y, Kim DS, Park SH. Medial Epicanthal Fold Correction Using a Y-W Epicanthoplasty in Asian Eyelids. J Craniofac Surg. 2024.

[70] Chen J, Zhang J, Xi W, Chen W, Yang F. The five-step medial epicanthoplasty: simple and standardized. BMC Ophthalmol. 2025;25:292.

[71] Liu N, Zhang J, Song N. Skin Replacement Epicanthoplasty and Concomitant Septoaponeurosis Junctional Thickening Double Eyelidplasty for Cosmetic Blepharoplasty in Asians. J Craniofac Surg. 2025;36:e679-e82.

[72] Long L, Xie L, Zeng H, Lai Q, Liu J, Liao W. Modified inverted 'L' epicanthoplasty combined with incisional blepharoplasty for epicanthal folds and single eyelids: a clinical outcomes study. Am J Transl Res. 2025;17:2014-22.

[73] Wang S, Li T, Liu H, Zhang D, Sr. The Medial Canthus Fibrous Band's Impact on Epicanthal Fold Severity and Classification in Asians: Implications for Epicanthoplasty. Aesthet Surg J. 2024;44:580-7.

[74] Jung JH, Kim HK, Choi HY. Epiblepharon correction combined with skin redraping epicanthoplasty in children. J Craniofac Surg. 2011;22:1024-6.

[75] Sa HS, Lee JH, Woo KI, Kim YD. A new method of medial epicanthoplasty for patients with blepharophimosis-ptosis-epicanthus inversus syndrome. Ophthalmology. 2012;119:2402-7.

[76] Oh J, Lee K. Medial lower lid epiblepharon repair solely by skin-redraping medial epicanthoplasty. Br J Ophthalmol. 2014;98:1437-41.

[77] Ni J, Shao C, Wang K, Chen X, Zhou S, Lin H. Modified Hotz Procedure Combined With Modified Z-Epicanthoplasty Versus Modified Hotz Procedure Alone for Epiblepharon Repair. Ophthalmic Plast Reconstr Surg. 2017;33:120-3.

[78] Chen B, Liu J, Ni J, Zhou S, Chen X. Lower eyelid tension balance reconstruction: A new procedure for the repair of congenital epiblepharon with epicanthus. J Plast Reconstr Aesthet Surg. 2019;72:842-7.

[79] Choi JW, Gaxiola-Garcia MA, Kang MK, Kim SC, Jeong WS, Koh KS. Correction of Congenital Telecanthus by Extended Medial Epicanthoplasty With Skin Redraping Method. Ann Plast Surg. 2019;82:528-32.

[80] Hu SL, Shi WQ, Su T, Ge QM, Li QY, Li B, et al. Surgical correction of recurrent epiblepharon in Chinese children using modified skin re-draping epicanthoplasty. Int J Ophthalmol. 2021;14:217-22.

[81] Mimura M, Sato Y, Fujita Y, Korn BS, Kikkawa DO, Oku H, et al. Adjustable Medial Epicanthoplasty Using a Rotational Flap for Epiblepharon Repair. J Craniofac Surg. 2022;33:1218-21.

[82] Hu S, Li J, Lu Y, Zhao S, Shao Y. Minimal lower eyelid epicanthoplasty combined with thermal contraction to treat epiblepharon in chinese children. BMC Ophthalmol. 2023;23:18.

[83] Ma T, Xu L, Chen Y, Zhang J, Han X. Skin-redraping epicanthoplasty combined with the modified Hotz procedure to treat recurrent trichiasis in adults caused by congenital entropion. J Plast Reconstr Aesthet Surg. 2023;81:164-8.

[84] Medel R, Scherrer E, Castano Fernandez-Vega L, Racana JI, Cordero C, Panos Palacios MI, et al. Lambda-double-fixation for medial epicanthoplasty in Blepharophimosis - Ptosis - Epicanthus Inversus Syndrome. Orbit. 2024:1-7.

[85] Watanabe H, Uemura K, Sakata Y, Inada M, Nakanishi M, Yamada G, et al. Evaluation of epicanthoplasty for child epiblepharon. J Plast Reconstr Aesthet Surg. 2025;105:317-22.
